# The effects of a strength and neuromuscular exercise programme for the lower extremity on knee load, pain and function in obese children and adolescents: study protocol for a randomised controlled trial

**DOI:** 10.1186/s13063-015-1091-5

**Published:** 2015-12-23

**Authors:** Brian Horsak, David Artner, Arnold Baca, Barbara Pobatschnig, Susanne Greber-Platzer, Stefan Nehrer, Barbara Wondrasch

**Affiliations:** Department of Physiotherapy, St. Pölten University of Applied Sciences, St. Pölten, Austria; Department of Biomechanics, Kinesiology and Applied Computer Science, University of Vienna, Vienna, Austria; Department of Paediatrics and Adolescent Medicine, Medical University of Vienna, Vienna, Austria; Centre for Regenerative Medicine and Orthopaedics, Danube University Krems, Krems, Austria

**Keywords:** Gait analysis, Biomechanics, Knee-adduction moment, KAM, Physiotherapy, Obesity, Children

## Abstract

**Background:**

Childhood obesity is one of the most critical and accelerating health challenges throughout the world. It is a major risk factor for developing varus/valgus misalignments of the knee joint. The combination of misalignment at the knee and excess body mass may result in increased joint stresses and damage to articular cartilage. A training programme, which aims at developing a more neutral alignment of the trunk and lower limbs during movement tasks may be able to reduce knee loading during locomotion. Despite the large number of guidelines for muscle strength training and neuromuscular exercises that exist, most are not specifically designed to target the obese children and adolescent demographic. Therefore, the aim of this study is to evaluate a training programme which combines strength and neuromuscular exercises specifically designed to the needs and limitations of obese children and adolescents and analyse the effects of the training programme from a biomechanical and clinical point of view.

**Methods/Design:**

A single assessor-blinded, pre-test and post-test randomised controlled trial, with one control and one intervention group will be conducted with 48 boys and girls aged between 10 and 18 years. Intervention group participants will receive a 12-week neuromuscular and quadriceps/hip strength training programme. Three-dimensional (3D) gait analyses during level walking and stair climbing will be performed at baseline and follow-up sessions. The primary outcome parameters for this study will be the overall peak external frontal knee moment and impulse during walking. Secondary outcomes include the subscales of the Knee injury and Osteoarthritis Outcome Score (KOOS), frontal and sagittal kinematics and kinetics for the lower extremities during walking and stair climbing, ratings of change in knee-related well-being, pain and function and adherence to the training programme. In addition, the training programme will be evaulated from a clinical and health status perspective by including the following analyses: cardiopulmonary testing to quantify aerobic fitness effects, anthropometric measures, nutritional status and psychological status to characterise the study sample.

**Discussion:**

The findings will help to determine whether a neuromuscular and strength training exercise programme for the obese children population can reduce joint loading during locomotion, and thereby decrease the possible risk of developing degenerative joint diseases later in adulthood.

**Trial registration:**

ClinicalTrials NCT02545764, Date of registration: 24 September 2015.

**Electronic supplementary material:**

The online version of this article (doi:10.1186/s13063-015-1091-5) contains supplementary material, which is available to authorized users.

## Background

Childhood obesity is one of the most critical and accelerating global health challenges. It already affects 17 % of all children and adolescents in the United States [1], with the incidence rate consistently rising in the majority of countries around the world [2]. In the last 10 years the incidence of childhood obesity in Germany increased by 50 %, with one out of six children qualifying as obese. Overweight children and adolescents encounter a higher risk of remaining obese as adults [3]. Similar statistics can be found in Austria, where the incidence of overweight children increased by 6 % from 2008 to 2012 [4]. Elmadfa has reported [4], that in 2012, approximately 17 % of Austrian children were overweight and 7 % were obese. Increases in the prevalence and incidence rates are alarming as childhood obesity is closely associated with not only obesity in adulthood, but also with several biomechanical risks factors that may result in developing lower-extremity misalignments. Factors such as foot deformities [5], large knee varus/valgus angles and moments, and muscular dysfunction [6–8], due to lower extremity misalignment, often result in reduced physical activity from the accompanying pain and discomfort [6]. Additionally, the simultaneous combination of excess body mass and lower-extremity misalignments results in increased joint stresses and articular cartilage damage during locomotion [9], which consequently might increase the risk of knee osteoarthritis or other degenerative joint diseases [10, 11].

The most common site affected by degenerative joint disease is the medial tibio-femoral joint compartment [12]. During everyday activities, particularly during walking [13] and stair climbing [14], this joint bears approximately 60–80 % of the compressive loads in neutrally aligned knees [15]. Misalignment of the lower leg, in either the valgus or varus direction, has been found to negatively influence the distribution of loads across the tibio-femoral joint compartments [16]. Tetsworth et al. [16] observed that a 4–6 % increase in varus misalignment affects medial compartment loading by up to 20 %. Similarly, Shultz et al. [6] reported that varus alignment may lead to a higher risk of encountering exacerbated symptoms such as pain and discomfort due to the excessive load on the medial compartment. This is of particular concern as the risk of valgus misalignment is significantly increased during walking. The combination of joint misalignment and overweight results in lower velocity, cadence, and a greater step width due to distortion of the tarsus [6]. Furthermore, overweight and obese children show less knee and hip flexion during walking, which indicates a more rigid posture. Gushue et al. [11] quantified three-dimensional (3D) knee joint kinematics and kinetics during walking in children and adolescents of varying body mass. They found significant differences in knee flexion moments in children who were overweight during walking and also reported a significant increase in frontal knee moments during the early stance phase. Gushue et al. [11] concluded, that while overweight children may develop a gait adaption to maintain similar knee extensor loads, they are unable to compensate for alterations in the frontal plane that consequently may lead to increased medial compartment loads. These findings strongly complement the results by Shultz et al. [17], who reported that overweight children experienced increased joint moments at the hip, knee and ankle joints compared to healthy controls. Shultz et al. [17] suggested that there is a strong relationship between absolute increased peak joint moments and the risk of unfavourable joint loading, skeletal misalignment, and injury in obese children. Moreover, the authors found that significant differences were eliminated between groups for peak joint moments when body mass was included as a covariate. These results illustrate the impact excess mass has on the absolute amount of force applied to the joint. McMillan et al. [18] stated that the repetitive stresses on the knee joint structures, related to the greater frontal plane excursion and moments during stance, are critical due to the potential for damage to knee joint structures, pain, limited motion, and resultant disabilities. Recent investigations have observed that these effects of excess body mass are also present in the biomechanics of walking upstairs [14]. Strutzenberger et al. [14] concluded that alterations in joint moments due to excess body mass, contribute to a cumulative overloading of the joints through adolescence, and potentially result in a greater risk of developing premature knee and hip osteoarthritis. The studies cited above indicate that obese children encounter a serious risk of developing unfavourable gait patterns and, therefore, have an increased expectancy of experiencing greater joint loads during locomotion.

In clinical gait biomechanics knee joint loading is often estimated by using the external frontal knee moment (e.g. external knee abduction or adduction moment) and impulse as surrogate parameters [19, 20]. While peak external frontal knee moments only measure load at one instance of stance, the external frontal knee moment impulse includes not only the load magnitude, but also the duration of stance, thus providing a more comprehensive measure [21].

Recently, Huang [22] evaluated differences in gait biomechanics between obese and average-weight children and also analysed the effects of weight loss and muscle strength training on gait characteristics via musculoskeletal modelling and simulation techniques. Huang observed that both weight loss and muscle strength training led to positive changes in kinematics and kinetics in gait characteristics. However, this study only analysed gait characteristics during level walking.

Even though a large number of guidelines exist for muscle strength training and neuromuscular training programmes [23], they are not specifically designed for the obese children and adolescent target groups. Furthermore, to the best of our knowledge, there is still a substantial lack of well-designed studies examining the effects of such interventions from both a biomechanical and clinical perspective. Therefore, the aim of this study is threefold: (1) to evaluate a training programme that combines strength and neuromuscular exercises. All aspects of the programme will be specifically designed to the needs and limitations of obese children and adolescents; (2) to examine if this training programme can positively affect lower-extremity joint loads during walking and stair climbing as well as knee-related ratings in well-being and function. Based on aim number (2) the two primary research hypotheses are as follows:

- H1: the external frontal knee moment and impulse during walking will be reduced by the training programme.
- H2: the training programme will improve self-reported knee function and knee-related well-being.

Aim number (3) is to evaluate the training programme from a clinical and health status perspective, including additional biomechanical analyses (e.g. altered movement strategies during walking and stair climbing) and cardiopulmonary exercise testing that will be used to quantify aerobic fitness effects; anthropometric measures, nutritional status and psychological status will be used to characterise the study sample.

## Methods/Design

### Trial design

**Fig. 1:**
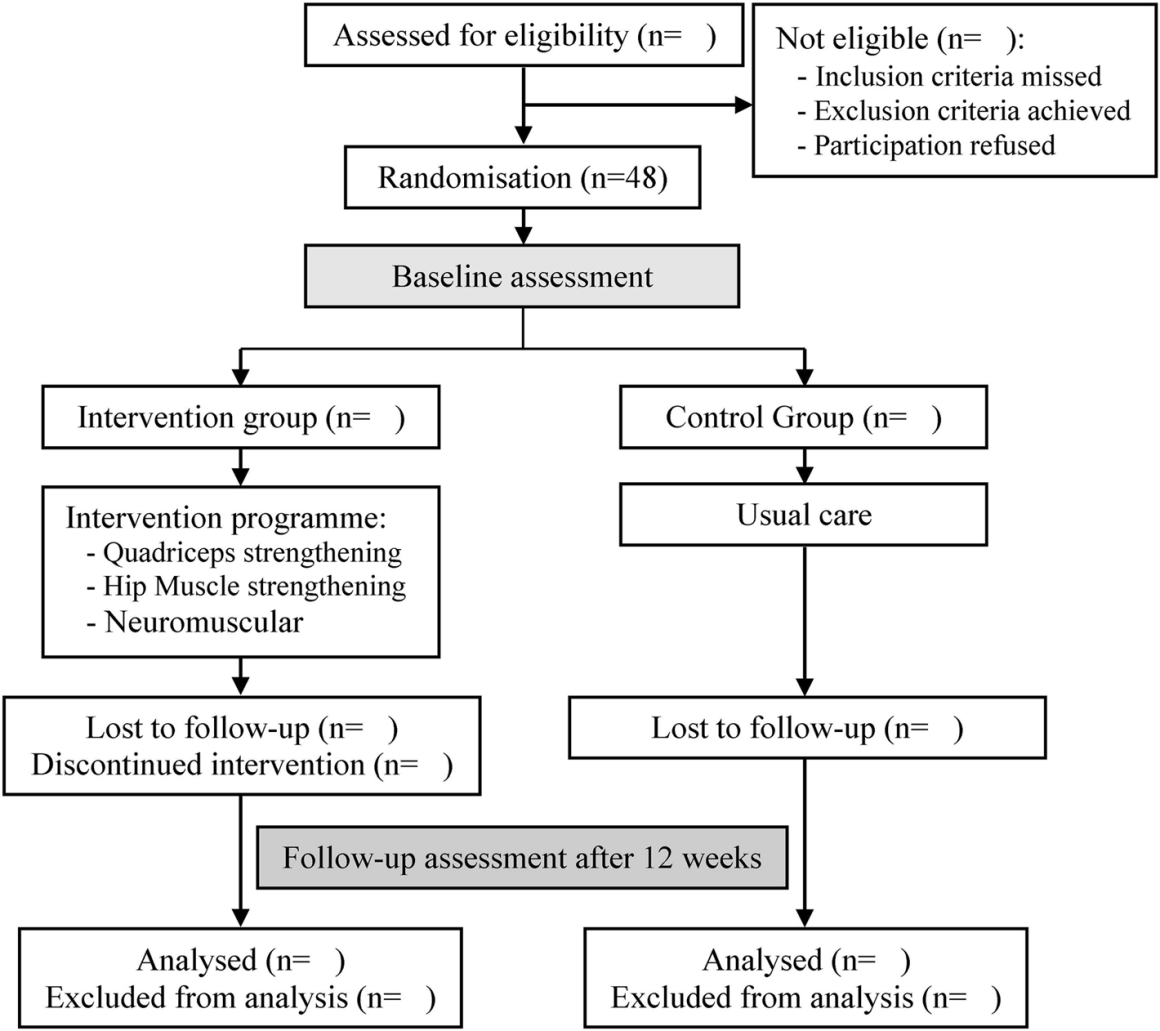
Flow diagram of the progress through the phases of the trial

This study will be a single assessor-blinded, pre-post-test randomised controlled trial, which conforms to Consolidated Standards of Research Trials (CONSORT) guidelines for non-pharmacological studies [24], with one intervention group (IG) and one control group (CG). A flowchart of the trial design is illustrated in Fig. 1. Measurements will be performed close at the start of the intervention programme (baseline assessment) and immediately after the 12-week intervention (follow-up assessment). Baseline and follow-up measurements will be performed by the same assessors, who will be blinded for both groups.

### Study population

A total of 48 obese boys and girls aged between 10 and 18 years will be recruited for this study. Sample size estimation revealed that 48 participants is the minimum number which will guarantee that the study is not underpowered. Nevertheless, depending on time and other resources available, we will focus on achieving more than the planned sample size. Participants will be recruited from the community in metropolitan Vienna, Austria. For recruitment purposes the study will be advertised at an outpatient clinic for paediatrics. Participants will be eligible if the following inclusion criteria are met:

1. Male or female
2. Age: 10–18 years
3. Body mass index (BMI) greater than the 97th percentile [25]
4. Availability: can participate in two exercises session per week for a period of 12 weeks

Participants will be excluded if one or more of the following criteria are met:

1. Present syndromes associated with obesity (e.g. Prader-Willi syndrome and similar disorders)
2. Chronic joint diseases, osteoarthritis surgery
3. Neuro-motor diseases

All adverse and serious adverse reactions will be documented, monitored and participants will be discontinued based on the decision of the investigator.

### Randomisation and allocation concealment

All eligible participants will be consecutively randomised to either the control or intervention group. Randomisation will be performed using a randomisation program and consecutively numbered, sealed and opaque envelopes containing allocation information. These will be prepared by a co-investigator who is uninvolved in participant testing or training.

### Ethical aspects

This study was approved by the ethics committee of the Medical University of Vienna (Ethics number: 1445/2013). Informed consent will be obtained from all participants and their legal representatives prior to the study by the primary investigator.

### Intervention programme

Intervention group participants will undergo a specialised 12-week training programme. An additional file shows the training programme in more detail (see Additional file 1). This programme, consisting of 45–60 minute training sessions, will be performed twice a week and will be supervised by physiotherapists. The training programme will consist of muscular strength and neuromuscular training exercises focusing on hip abductor and quadriceps muscles. Strengthening of the quadriceps and hip muscles in combination with neuromuscular exercises aims to improve the position of the knee in relation to the hip and the ankle joints during locomotion. It may, therefore, enhance activation of muscle groups generating internal moments which counteract the external moments in the frontal plane of the knee joint.

The first training session will be a familiarisation session [26], wherein participants will receive general information about the exercise programme (organisation, availability of the physiotherapist, training location) and an introduction about the rationale for this programme. Additionally, participants will receive information regarding basic principles of exercise performance. The specific training programme will follow state of the art recommendations in musculoskeletal rehabilitation [27–29].

#### Quadriceps strengthening

Participants will perform both non-weight-bearing and weight-bearing exercises. It has been observed that weight-bearing exercises may increase joint loading and may, therefore, provoke symptoms such as pain and effusion in patients with knee disorders [30, 31]. The participants will begin with three non-weight-bearing exercises [27] to become familiarised with the programme. After four weeks, three weight-bearing exercises will be additionally implemented. Based on the quadriceps training protocol of Lim et al. [28], ten repetitions will be performed in each set of quadriceps exercises beginning with two sets and progressing as quickly as possible to three sets. The starting weight will be determined by asking the participants about their level of effort, which should be 5–8 (out of 10) on the modified Borg rated perceived exertion (RPE) CR-10 scale [32]. During all exercises the self-perceived level of effort should be 5 (out of 10) on a RPE CR-10 scale for strength training. If pain occurs, resistance, frequency or number of repetitions will be reduced. The quadriceps strengthening exercises are summarised in Table 1.

**Table 1:** Quadriceps strengthening exercises

Exercise
1. Straight leg raise in supine position, raise leg to 30° of hip flexion using resistance from ankle weights
2. Small arc knee extension with a roll under the knee using resistance from ankle weights
3. Full knee extension in sitting position starting from 90° of knee flexion using resistance from ankle weights
4. Small arc squats (from full extension to 30° of knee flexion) on both legs with a ball placed against a wall using two dumbbells, one in each hand
5. Small arc squats (between 40° and 90° of knee flexion) on both legs with a ball placed against a wall using two dumbbells, one in each hand
6. Step up on a ‘stepper’ (height: 30 cm)

#### Hip muscle strengthening

For hip muscle strengthening the exercise programme reported by Bennell et al. [29] will be used. This programme includes six exercises addressing the hip abductor and adductor muscles. Participants will perform three sets with ten repetitions. The intensity of exercises will be adjusted according to the participant´s ability to complete ten repetitions. All hip muscle strengthening exercises are summarised in Table 2.

**Table 2:** Hip strengthening exercises

Exercise	
1. Abduction in sidelying: unilateral hip abduction performed in sidelying with the use of ankle cuff weights	
2. Abduction in standing: unilateral hip abduction performed standing with the use of a resistance band	
3. Standing wall isometric hip abduction: performed in unipedal stance with the opposite limb in 90° of knee flexion.	
4. Clam in sidelying position with the resistance of an elastic band	
5. Bridging bilateral	
6. Bridging unilateral with the opposite limb in approximately 90° of knee flexion.	

#### Neuromuscular exercises

The neuromuscular exercises consist of one-legged and two-legged balance exercises. Based on the protocol by Bennell et al. [29] participants will be instructed to improve control of knee and hip muscles by practicing more neutral knee positioning during specific tasks [29]. The quality of performance is critical and participants should aim to keep their knee over the foot during the exercises. Furthermore, knee flexion should not exceed 30° to avoid high loading within the joint and to minimise pain. To insure progression, unstable surfaces such as a foam mat or balance board will be provided. Additionally, repetitions will be increased and accompanied by varying movement directions and exercise velocities. The progression will be based on individual demands so that pain during exercises may be minimised. The neuromuscular exercises are summarised in Table 3.

**Table 3:** Neuromuscular exercises

Exercise	
1. Bilateral stance on a soft surface (progression: foam mat and wobble board)	
2. Bilateral stance on a soft surface (progression: foam mat and wobble board) with eyes closed	
3. Bilateral stance on a soft surface (progression: foam mat and wobble board ): two participants facing each other and passing a ball back and forth	
4. Squat lunge static on a soft surface (progression: foam mat and wobble board)	
5. Squat lunge static on a soft surface and throwing and catching a ball	
6. Unilateral stance on a soft surface (progression: foam mat and wobble board)	
7. Unilateral stance of a soft surface (progression: foam mat and wobble board) with eyes closed	
8. Two participants are standing on a soft surface with both legs (progression: foam mat and wobble board) facing each other and passing a ball back and forth	

### Outcome parameters

#### Gait analysis

To assess dynamic loading of the knee during walking and stair ascent and descent all participants will undergo a 3D gait analysis at self-selected and controlled walking speed. Kinetic data will be collected using two force plates (KISTLER, Winterthur, Switzerland) at a sampling rate of 1000 Hz each, which will be placed level with the ground. Kinematic data will be collected using a motion capture system (VICON, Oxford, UK), comprised of eight infrared-cameras at a sampling rate of 150 Hz and time-synchronised to the force plates. The participants will be asked to walk several times along a 12-m walkway without any further instructions, while walking speed will be monitored using photo sensors. Afterwards, the mean walking speed will be determined and used for all upcoming analyses as the reference walking speed, allowing for a tolerance of approximately ±0.2 m/s. The experimental staircase will consist of three steps with a dimension of 16 cm height and 30 cm depth, and a width of 80 cm. The first step is directly mounted to a force plate level with the ground. For stair ascent, the initial step before stepping onto the staircase as well as the first step onto the stair case will be captured by both force plates. For stair descent, contact at the last staircase and the first ground contact will be captured. For the stair climbing trials participants will be asked to ascend and descend the stairs, placing only one foot on each step with a cadence of approximately 110 steps per minute using a metronome [14]. The order of test condition (walkway and stair climbing) will be randomised for all participants to avoid order effects.

From the kinematic data linear and angular positions, velocities and accelerations will be derived. For this purpose the Modified Cleveland Clinic Marker Set will be used. Inverse dynamics techniques will be used to determine resultant joint moments for hip and knee joints in sagittal and frontal plane. All data will be expressed in percentage of gait cycle. Resultant joint moments will be normalised for body weight (BW) × height (HT) and expressed as Nm/(BW × HT)%. The ground reaction force will be normalised to percent of body weight (%BW).

The primary outcome parameters will be the overall peak external frontal knee moment normalised for BW × HT (Nm/(BW × HT)%) and the external frontal knee moment impulse (see Table 4). Additionally, external joint moments for the sagittal and frontal plane for hip, knee and ankle joints as well as spatio-temporal parameters will also be assessed [33].

**Table 4:** Summary of outcome measures

Primary outcome parameters	Instrument for data collection
Overall peak external frontal knee moment and impulse	3D gait analysis during walking
Secondary outcome parameters	
Frontal and sagittal kinematics and kinetics for hip, knee and ankle joints	3D gait analysis during walking and stair climbing
KOOS subscales (Austrian-German version)	Knee function in daily living (ADL)
	Knee function in sport and recreation (Sport/Rec)
	Knee-related quality of life (QOL)
	Knee pain and other symptoms
Changes of function and strength of the targeted muscle groups	Physical examination/orthopaedic status
Adherence to training programme	Percentage of completed sessions among the number of intended exercise sessions
Ratings of knee-related pain	7-point ordinal scale (followed for the duration of the intervention)
Other outcome parameters	
Cardiopulmonary testing	Symptom-limited cardiopulmonary exercise testing on a cycle ergometer
Anthropometry	Anthropometric measurements
Body composition	Bioimpedance analysis
Nutritional status	24-hour recall method (daily, self-reported consumption of food-intake)
Psychological status	AD-EVA test inventory, Child Behaviour Checklist (CBCL/4-18 )
Blood samples	Growth hormones (GH, IGFBP3, AP) and inflammation (procalcin, TGF-α, IL-8) from venous blood samples
All measures will be recorded at baseline and follow-up unless stated otherwise	

*3D* three-dimensional, *ALS*, *CRP* C-reactive protein, *GH* growth hormone, *IGF*-*1* insulin-like growth factor-1, *IGFB3* insulin-like growth factor binding protein 3, *IL*-*6* interleukin-6, *KOOS* Knee injury and Osteoarthritis Outcome Score, *TGF* Transforming growth factor, *AP* Anterior pituitary hormone

#### Orthopaedic status

An orthopaedic status, which includes the assessment of posture and joint function of the upper and lower extremities of each participant, will be determined [34]. An instructed orthopaedist will inspect the participants at baseline as well as at the follow-up assessment.

#### Physical examination

The Austria-German version of the Knee Injury and Osteoarthritis Outcome Score (KOOS) questionnaire will be used to assess the participants’ opinion about their knee problems [35–37]. The questionnaire is valid, reliable and was compared to other instruments in previous studies [36, 38–40]. This questionnaire consists of five subscales: knee pain and other symptoms, function in daily living (ADL), function in sport and recreation (Sport/Rec) and knee-related quality of life (QOL). The scores of the subscales of KOOS will be used as secondary outcome measures as recommended by the KOOS manual [41]. Changes of function and strength of the targeted muscle groups will also be assessed. Therefore, a hand-dynamometer to investigate differences in muscle strength of the quadriceps and hip abductor muscles of both legs before and after the intervention programme will be used [42].

#### Anthropometry and body composition

Anthropometric and body composition variables will be assessed to quantify size and shape of the participants by using conventional anthropometry and bioimpedance analysis (BIACORPUS RX 4000, Medical Health Care GmbH, Karlsruhe, Germany; Tanita scale, Type BC 418MA, Tokyo, Japan). The anthropometric evaluation will include the measurement of height, weight, waist circumference, hip circumference, waist-to-hip ratio, and calculation of the body mass index (weight/height^2^ in kg/m^2^). Body composition parameters will include, fat mass (FM), fat free mass (FFM), total body water (TBW), body cell mass (BCM), extracellular mass (ECM) and lean body mass (LBM).

#### Cardiopulmonary exercise testing

Obese children and adolescents frequently show a reduced aerobic performance that can be improved by increasing physical activity [43]. Therefore, the influence of the training programme on aerobic fitness will be assessed by submitting the participants to a symptom-limited cardiopulmonary exercise testing on a cycle ergometer [44]. Expired gases will be continuously collected (Jaeger®,Wuerzburg, Germany) throughout testing, thereby calculating the following parameters: anaerobic threshold (AT), maximum power (P), ventilatory threshold (VT2) and peak oxygen consumption (pVO_2_).

#### Blood samples

Quantification of routine blood sampling, growth hormones (growth hormone (GH), insulin-like growth factor binding protein 3 (IGFBP3), anterior pituitary hormone (AP)) and inflammation (procalcin, transforming growth factor alpha (TGF-α), interleukin-8 (IL-8)) will be performed from venous blood samples. All obese patients will follow the routine blood measurements in the outpatient clinic. 

#### Psychological and nutritional status

The AD-EVA test inventory, an interdisciplinary test system for diagnosis and evaluation of obesity and other illnesses influenced by eating habits and motoric behaviour, as well as the Child Behaviour Checklist (CBCL/4-18) will be used to determine the psychological status of the participants. The CBCL is a widely used method to identifying problem behaviour in children [45], whereas the AD-EVA inventory is used to analyse psychological variables which are considered to cause or promote obesity [46]. The nutritional status will be assessed using the 24-hour recall method. This method records the daily, self-reported consumption of food-intake. In combination with a standardised nutritional food programme (EBISpro, Zurich, Switzerland) the intake of macronutrients and micronutrients will be estimated.

#### Adherence to the training programme

Intervention group participants will be asked to keep a day-by-day exercise log including adherence and average overall pain during and after each exercise session. The physiotherapist supervising the intervention programme will also monitor adherence to the group sessions. Adherence will be considered as the percentage of actually completed sessions during the intervention period among the number of intended exercise sessions. Studies investigating the feasibility of different exercise programmes for musculoskeletal disorders, which show good outcomes for patients, typically reported adherence percentages of greater than 80 % [26, 47, 48]. Therefore, we will a priori consider an adherence percentage of 85 % as feasible for our exercise programme. In addition, participants will be asked about their rating of change in knee-related well-being, pain and joint function using a 7-point ordinal scale.

### Sample size estimation

Primary outcome parameters for this study are the external frontal knee moment and impulse during walking. Recent studies have determined that a specific non-invasive training can facilitate a reduction of external knee adduction moment (EKAM) peaks of 13 % during stance [49]. In accordance with assumptions of Kean et al. [21], and the fact that EKAM impulse seems to be a more sensitive measure than peak EKAM [21], a magnitude of 5–10 % change in EKAM and external knee adduction moment impulse (EKAMI) seems to be achievable in this study. Based on our assumptions and the estimates of Kean et al. [21], we assume a medium to large effect size of *d* = 0.45. Based on a two-tailed repeated comparison of two groups (IG and CG) using one covariate (weight loss) for repeated analysis of covariance (ANCOVA), when the effect size is *d* = 0.45, power is 0.8, type I error is 0.05 and *df* = 1, the estimated total sample size is approximately 40. Allowing a dropout rate of approximately 20 % yields a total sample size of 48. Sample size was estimated using G*Power 3.1.3 [50, 51].

### Statistical analysis

Data will be analysed and processed in MATLAB (The MathWorks Inc., Natick, MA, USA). Statistical analysis will be conducted using IBM SPSS Statistics 23 or greater (IBM Corporation, Armonk, NY, USA). The level of significance will be set a priori to 0.05 for all analyses. Data will be generally inspected for normal distribution using the Kolmogorov-Smirnov test as well as skewness and kurtosis parameters. Data (e.g. demographic and baseline data) will be summarised by descriptive statistics using the mean (SD) or the median (IR). All data will be analysed on the basis of an intention-to-treat (ITT) analysis as well as per protocol (PP). Repeated measures ANCOVA will be used to compare both groups in primary and secondary outcome parameters using both time and group by time interactions [52]. Weight loss of participants will be used as a covariate to account for a possible weight reduction during the entire training programme. Weight loss was identified as an important covariate as a recent study has revealed that reduction of weight in adolescents might lead to a 12 % decrease in knee joint loads [53]. For non-normal continuous data the Mann-Whitney *U* test or the Kruskal-Wallis signed rank test will be used.

## Discussion

This study will attempt to evaluate a 12-week strength training and neuromuscular exercise programme for obese children from a biomechanical and clinical perspective. Effects on biomechanical gait characteristics during level walking and stair climbing will be analysed. Up to date only a few interventional studies exist: e.g. [22], which have analysed the impact of a training programme on gait during level walking of obese participants. This study aims at extending current research as well as adding analyses of changes in biomechanical gait characteristics during stair ascent and descent.

The main aspects of the programme focus on strengthening quadriceps and hip muscles in combination with neuromuscular exercises. The training programme aims at developing a more neutral alignment of the trunk and lower limbs during locomotion, thereby intending to improve the position of the knee in relation to the hip and the ankle joints. It may, therefore, enhance activation of muscle groups generating internal moments which counteract external moments in the frontal plane of the knee. The training programme will follow state of the art recommendations in musculoskeletal rehabilitation [27–29] and intends to motivate children and adolescents to exercise regularly, due to its specifically adjusted and attractive design for the age group in question. Further, only cost-effective equipment will be used, thus increasing its feasibility in common physiotherapy practices.

A single assessor-blinded, pre-test and post-test randomised controlled trial design will be utilised to investigate any biomechanical and clinical changes that may occur due to the intervention programme. The primary biomechanical outcome parameters in this study will be the overall peak external frontal knee moment normalised for BW × HT (Nm/(BW × HT)%) and the impulse. These parameters are well-accepted surrogate measures for knee joint loading [20]. Kutzner et al. [20] reported a high correlation between the tibio-femoral force measured by an instrumented knee implant and the EKAM (*R*^2^ = 0.76), suggesting that EKAM is well-suited to predict the medial tibio-femoral contact force, particularly during the early stance phase. Even though EKAM is a well-accepted measure for knee joint loading, concerns have recently emerged stating that peak EKAM only measures the load at one instance of stance and does not reflect joint loading over the entire stance phase [21]. Although peak moments can be reduced through slower walking speed, the increased duration of stance phase and, therefore, the time under load can result in an overall increase of joint loading. For this reason the impulse (e.g. external knee adduction moment impulse) has been demonstrated to provide a more comprehensive measure [21] as it takes into account both the magnitude of load and the duration of stance.

Clinical outcomes comprise the KOOS questionnaire, measures of self-reported pain during and after each training session and adherence to the training programme. In detail the KOOS questionnaire will assess participants’ knee pain and other symptoms, function in daily living (ADL), function in sport and recreation (Sport/Rec) and knee-related quality of life (QOL). In addition, the training programme will be evaluated from a clinical perspective by including the following analysis: cardiopulmonary testing to quantify aerobic fitness effects as well as anthropometric measures, nutritional and psychological status.

The primary findings of this study will help to determine whether a neuromuscular and strengthening exercise programme for an obese population of children can reduce joint loading during locomotion, and thus decrease the possible risk of developing degenerative joint diseases in adulthood.

## Trial status

The ethics committee has approved the study protocol. Participant recruitment will start at the end of September 2015 and it is anticipated that the necessary number of participants will be tested until the beginning of 2017. Data analysis will be conducted subsequently.

## Consent

Written informed consent was obtained from the patient(s) for publication of this manuscript and accompanying images. A copy of the written consent is available for review by the editor-in-chief of this journal.

## Additional file

Additional file 1:
**Training programme.** Image series of hip, quadriceps and neuromuscular training programme showing start and end position of each exercise. (PDF 1055 kb)

## Abbreviations

3D: three-dimensiona
ADL: function in daily living (KOOS)
AP: anterior pituitary hormone
ANCOVA: analysis of covariance
AT: anaerobic threshold
BCM: body cell mass
BMI: body mass index
BW: body weight
CBCL/4-18: Child Behaviour Checklist
CG: control group
CONSORT: Consolidated Standards of Research Trials
ECM: extracellular mass
EKAM: external knee adduction moment
EKAMI: external knee adduction moment impulse
FFM: free fat mass
FM: fat mass
GH: growth hormone
HT: height
IG: intervention group
IGFBP: insulin-like growth factor binding protein
IL: interleukin
IR: interquartile range
ITT: intention-to-treat
KOOS: Knee injury and Osteoarthritis Outcome Score
LBM: lean body mass
P: maximum power
PP: per protocol
pVO_2_: peak oxygen consumption
QOL: quality of life (KOOS)
RPE: rated perceived exertion
SD: standard deviation
Sport/Rec: function in sport and recreation (KOOS)
TBW: total body water
VT2: ventilatory threshold
TGF-α: transforming growth factor alpha

## References

[1] Ogden CL, Carroll MD, Kit BK, Flegal KM (2014). Prevalence of childhood and adult obesity in the United States, 2011–2012. JAMA.

[2] Flodmark C-E, Lissau I, Moreno LA, Pietrobelli A, Widhalm K (2004). New insights into the field of children and adolescents’ obesity: the European perspective. Int J Obes.

[3] de Sa Pinto AL, de Barros Holanda PM, Radu AS, Villares SM, Lima FR (2006). Musculoskeletal findings in obese children. J Paediatr Child H.

[4] Elmadfa I (2012). Österreichischer Ernährungsbericht 2012.

[5] García-Rodríguez A, Martín-Jiménez F, Carnero-Varo M, Gómez-Gracia E, Gómez-Aracena J, Fernández-Crehuet J (1999). Flexible flat feet in children: a real problem?. Pediatrics.

[6] Shultz SP, Anner J, Hills AP (2009). Paediatric obesity, physical activity and the musculoskeletal system. Obes Rev.

[7] Henderson RC (1992). Tibia vara: a complication of adolescent obesity. J Pediatr.

[8] Shultz SP, D’Hondt E, Fink PW, Lenoir M, Hills AP (2014). The effects of pediatric obesity on dynamic joint malalignment during gait. Clin Biomech.

[9] Messier SP, Ettinger WH, Doyle TE, Morgan T, James MK, O’Toole ML (1996). Obesity: effects on gait in an osteoarthritic population. J Appl Biomech.

[10] Shultz SP (2008). Lower extremity biomechanical assessment of overweight and normal-weight children during self-selected and fast walking speeds.

[11] Gushue DL, Houck J, Lerner AL (2005). Effects of childhood obesity on three-dimensional knee joint biomechanics during walking. J Pediatr Orthoped.

[12] Ledingham J, Regan M, Jones A, Doherty M (1993). Radiographic patterns and associations of osteoarthritis of the knee in patients referred to hospital. Ann Rheum Dis.

[13] Bovi G, Rabuffetti M, Mazzoleni P, Ferrarin M (2011). A multiple-task gait analysis approach: kinematic, kinetic and EMG reference data for healthy young and adult subjects. Gait Posture.

[14] Strutzenberger G, Richter A, Schneider M, Mündermann A, Schwameder H (2011). Effects of obesity on the biomechanics of stair-walking in children. Gait Posture.

[15] Schipplein OD, Andriacchi TP (1991). Interaction between active and passive knee stabilizers during level walking. J Orthop Res.

[16] Tetsworth K, Paley D (1994). Malalignment and degenerative arthropathy. Orthop Clin North Am.

[17] Shultz SP, Sitler MR, Tierney RT, Hillstrom HJ, Song J (2009). Effects of pediatric obesity on joint kinematics and kinetics during 2 walking cadences. Arch Phys Med Rehab.

[18] McMillan AG, Pulver AME, Collier DN, Williams DSB (2010). Sagittal and frontal plane joint mechanics throughout the stance phase of walking in adolescents who are obese. Gait Posture.

[19] Birmingham TB, Hunt MA, Jones IC, Jenkyn TR, Giffin JR (2007). Test-retest reliability of the peak knee adduction moment during walking in patients with medial compartment knee osteoarthritis. Arthritis Rheum.

[20] Kutzner I, Trepczynski A, Heller MO, Bergmann G (2013). Knee adduction moment and medial contact force – facts about their correlation during gait. PLoS One.

[21] Kean CO, Hinman RS, Bowles KA, Cicuttini F, Davies-Tuck M, Bennell KL (2012). Comparison of peak knee adduction moment and knee adduction moment impulse in distinguishing between severities of knee osteoarthritis. Clin Biomech.

[22] Huang L (2014). The implications of childhood obesity on the musculoskeletal and locomotor systems: biomechanical analyses and exercise intervention. Department of Sport and Exercise Science Faculty of Science.

[23] Kristensen J, Franklyn-Miller A (2012). Resistance training in musculoskeletal rehabilitation: a systematic review. Brit J Sport Med.

[24] Boutron I, Moher D, Altman DG, Schulz KF, Ravaud P (2008). Extending the CONSORT Statement to randomized trials of nonpharmacologic treatment: explanation and elaboration. Ann Intern Med.

[25] Barlow SE, the Expert Committee (2007). Expert committee recommendations regarding the prevention, assessment, and treatment of child and adolescent overweight and obesity: summary report. Pediatrics.

[26] Mazières B, Thevenon A, Coudeyre E, Chevalier X, Revel M, Rannou F (2008). Adherence to, and results of, physical therapy programs in patients with hip or knee osteoarthritis. Development of French clinical practice guidelines. Joint Bone Spine.

[27] Lin D-H, Lin C-HJ, Lin Y-F, Jan M-H (2009). Efficacy of 2 non-weight-bearing interventions, proprioception training versus strength training, for patients with knee osteoarthritis: a randomized clinical trial. J Orthop Sport Phys.

[28] Lim B, Hinman RS, Wrigley TV, Sharma L, Bennell KL (2008). Does knee malalignment mediate the effects of quadriceps strengthening on knee adduction moment, pain, and function in medial knee osteoarthritis? A randomized controlled trial. Arthritis Rheum.

[29] Bennell KL, Egerton T, Wrigley TV, Hodges PW, Hunt M, Roos EM (2011). Comparison of neuromuscular and quadriceps strengthening exercise in the treatment of varus malaligned knees with medial knee osteoarthritis: a randomised controlled trial protocol. BMC Musculoskelet Disord.

[30] Baliunas AJ, Hurwitz DE, Ryals AB, Karrar A, Case JP, Block JA (2002). Increased knee joint loads during walking are present in subjects with knee osteoarthritis. Osteoarthr Cartilage.

[31] Lucchinetti E, Adams CS, Horton WE, Torzilli PA (2002). Cartilage viability after repetitive loading: a preliminary report. Osteoarthr Cartilage.

[32] Borg G, Ljunggren G, Ceci R (1985). The increase of perceived exertion, aches and pain in the legs, heart rate and blood lactate during exercise on a bicycle ergometer. Eur J Appl Physiol O.

[33] Benedetti M, Catani F, Leardini A, Pignotti E, Giannini S (1998). Data management in gait analysis for clinical applications. Clin Biomech.

[34] Fritsch P, Fritz M, Förster H, Gitter R, Kitzmüller E, Köstenberger M, et al. Sport- und Wettkampftauglichkeitsuntersuchungen im Kindes- und Jugendalter: Empfehlungen der Österreichischen Gesellschaft für Kinder- und Jugendheilkunde (ÖGKJ) und der Österreichischen Gesellschaft für Sportmedizin und Prävention (ÖGSMP). Monatsschrift Kinderheilkunde. 2015;163(10):1030-1036.

[35] Collins NJ, Misra D, Felson DT, Crossley KM, Roos EM (2011). Measures of knee function: International Knee Documentation Committee (IKDC) Subjective Knee Evaluation Form, Knee Injury and Osteoarthritis Outcome Score (KOOS), Knee Injury and Osteoarthritis Outcome Score Physical Function Short Form (KOOS-PS). Arthrit Care Res.

[36] Roos EM, Toksvig-Larsen S. Knee injury and Osteoarthritis Outcome Score (KOOS) – validation and comparison to the WOMAC in total knee replacement. Health Qual Life Out. 2003;1. 10.1186/1477-7525-1-17 PMC161802 12801417

[37] Roos EM, Lohmander LS (2003). The Knee injury and Osteoarthritis Outcome Score (KOOS): from joint injury to osteoarthritis. Health Qual Life Out.

[38] Roos EM, Roos HP, Lohmander LS, Ekdahl C, Beynnon BD (1998). Knee Injury and Osteoarthritis Outcome Score (KOOS) – development of a self-administered outcome measure. J Orthop Sport Phys.

[39] Roos EM, Roos HP, Ekdahl C, Lohmander LS (1998). Knee injury and Osteoarthritis Outcome Score (KOOS) – validation of a Swedish version. Scand J Med Sci Spor.

[40] Roos EM, Roos HP, Lohmander LS (1999). WOMAC Osteoarthritis Index – additional dimensions for use in subjects with post-traumatic osteoarthritis of the knee. Osteoarthr Cartilage.

[41] The 2012 User’s Guide to: Knee injury and Osteoarthritis Outcome Score KOOS. http://koos.nu/index.html. Accessed 01. 05. 2015.

[42] Ieiri A, Tushima E, Ishida K, Inoue M, Kanno T, Masuda T (2015). Reliability of measurements of hip abduction strength obtained with a hand-held dynamometer. Physiother Theory Pract.

[43] Vajda I, Mészáros J, Mészáros Z, Prókai A, Sziva A, Photiou A (2007). Effects of 3 hours a week of physical activity on body fat and cardio-respiratory parameters in obese boys. Acta Physiol Hung.

[44] Sietsema K, Wasserman K, Hansen JE, Sue DY, Stringer WW, Sun X-G (2011). Principles of exercise testing and interpretation: including pathophysiology and clinical applications.

[45] Achenbach TM, Rescorla L (2001). Manual for the ASEBA school-age forms & profiles: an integrated system of multi-informant assessment.

[46] Ardelt-Gattinger E, Meindl M (2010). Interdisziplinäres Testsystem Zur Diagnostik Und Evaluation Bei Adipositas Und Anderen Durch Ess-Und Bewegungsverhalten Beeinflussbaren Krankheiten (AD-EVA).

[47] Skou ST, Odgaard A, Rasmussen JO, Roos EM (2012). Group education and exercise is feasible in knee and hip osteoarthritis. Dan Med J.

[48] Steinhilber B, Haupt G, Miller R, Boeer J, Grau S, Janssen P (2012). Feasibility and efficacy of an 8-week progressive home-based strengthening exercise program in patients with osteoarthritis of the hip and/or total hip joint replacement: a preliminary trial. Clin Rheumatol.

[49] Haim A, Rubin G, Rozen N, Goryachev Y, Wolf A (2012). Reduction in knee adduction moment via non-invasive biomechanical training: a longitudinal gait analysis study. J Biomech.

[50] Erdfelder E, Faul F, Buchner A (1996). G*Power: a general power analysis program. Behav Res Meth Inst.

[51] Faul F, Erdfelder E, Lang A-G, Buchner A (2007). G*Power 3: a flexible statistical power analysis program for the social, behavioral, and biomedical sciences. Behav Res Methods.

[52] Field A (2009). Discovering statistics using SPSS: and sex and drugs and rock ‘n’ roll.

[53] Aaboe J, Bliddal H, Messier SP, Alkjær T, Henriksen M (2011). Effects of an intensive weight loss program on knee joint loading in obese adults with knee osteoarthritis. Osteoarthr Cartilage.

