# Supplementary material for: The effects of a strength and neuromuscular exercise programme for the lower extremity on knee load, pain and function in obese children and adolescents: study protocol for a randomised controlled trial
Source: Trials. 2015 Dec 23;16:586. doi: 10.1186/s13063-015-1091-5 (PMC4690219; doi:10.1186/s13063-015-1091-5)
Supplement: Additional file 1: — Training programme. Image series of hip, quadriceps and neuromuscular training programme showing start and end position of each exercise. (PDF 1055 kb) [file 13063_2015_1091_MOESM1_ESM.pdf]

Additional file 1

Exercise programme Part – Quadriceps strengthening

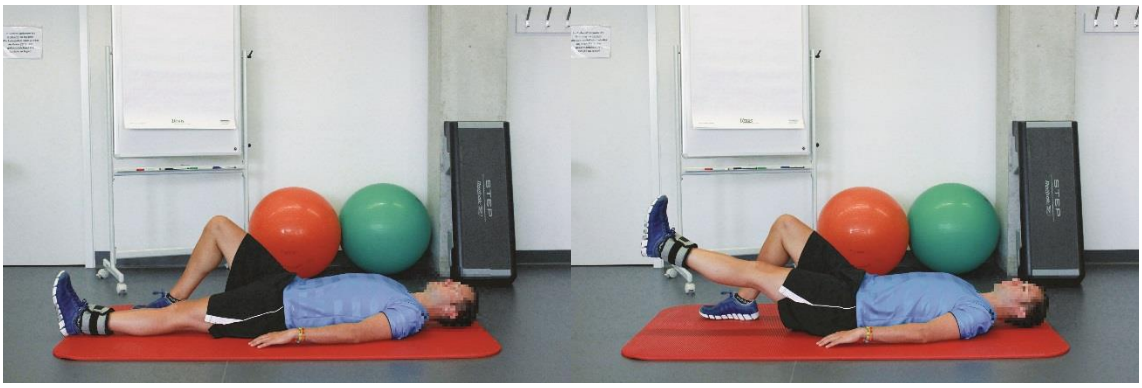

Knee 1: Straight leg raise in supine position using resistance of ankle weights

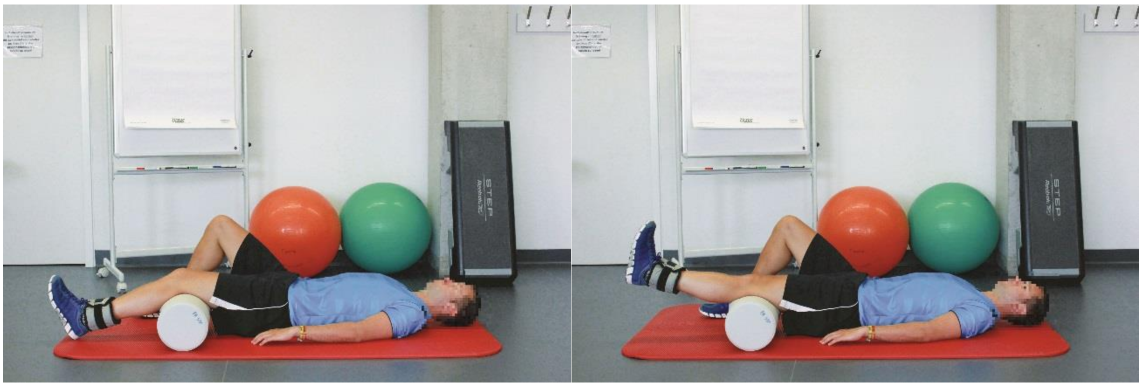

Knee 2: Small arc knee extension using resistance of ankle weights

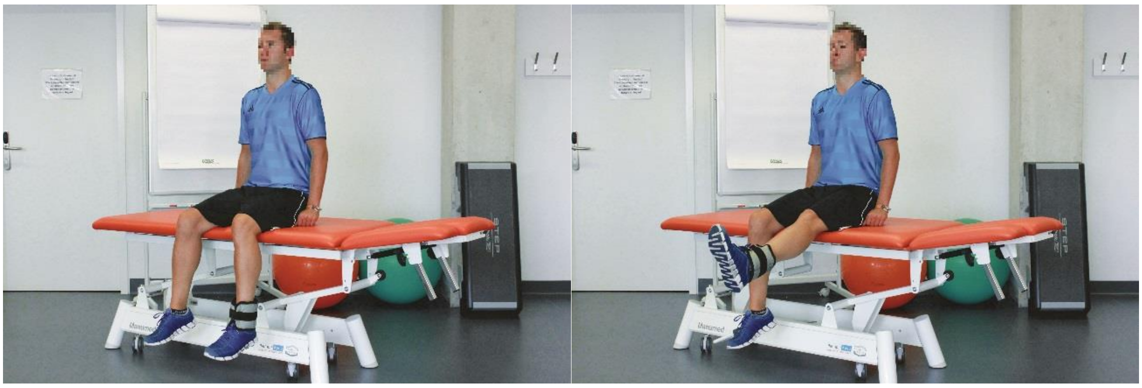

Knee 3: Full knee extension in sitting position

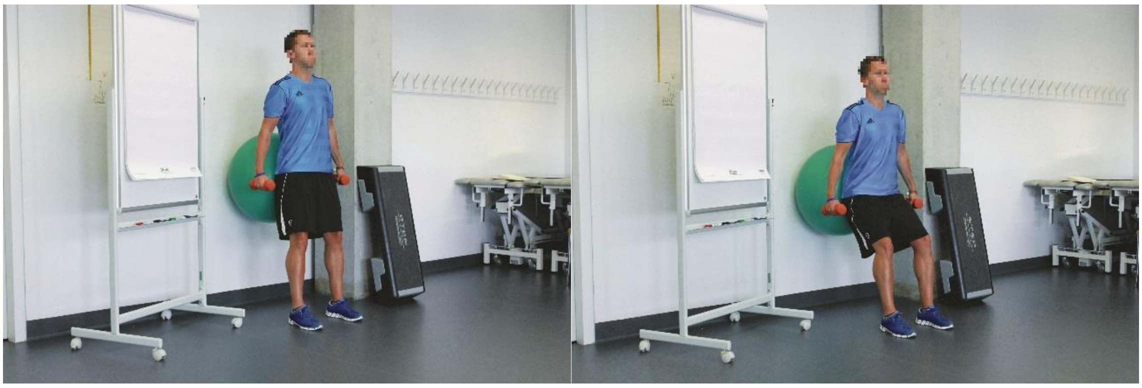

Knee 4: Small arc squats from 0-30

Additional file 1

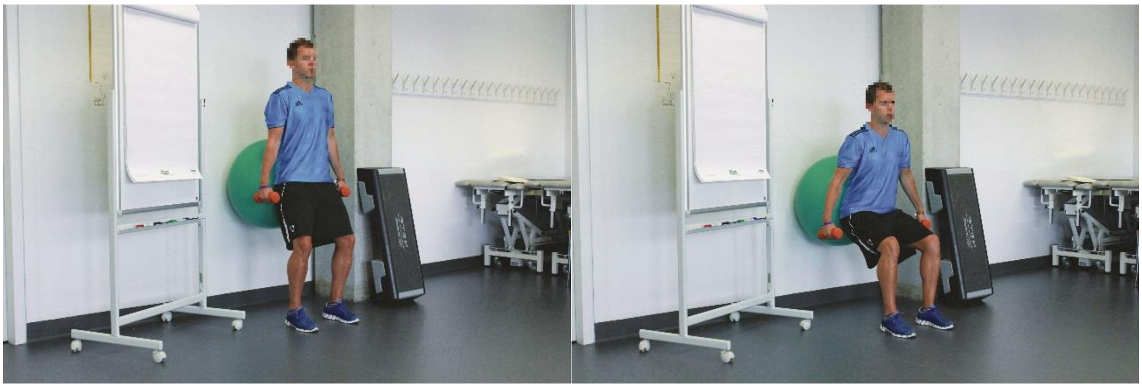

Knee 5: Small arc squats from 40-90

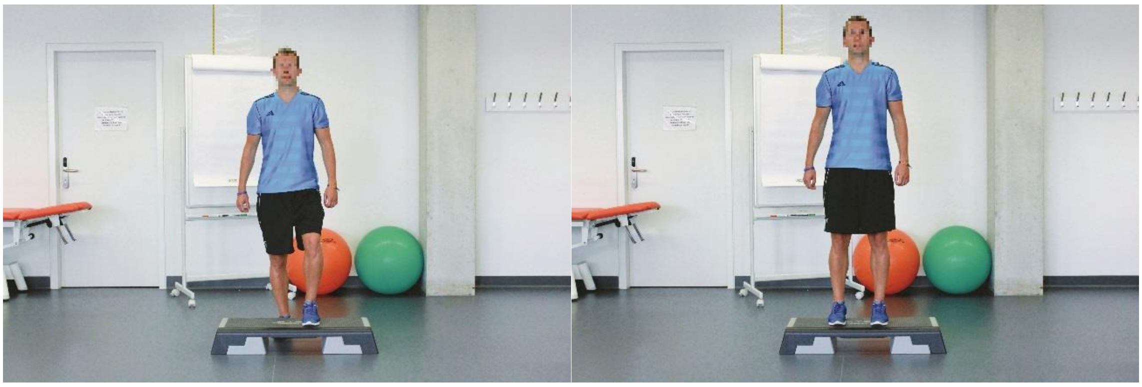

Knee 6: Step up

Additional file 1

Exercise programme Part - Hip strengthening

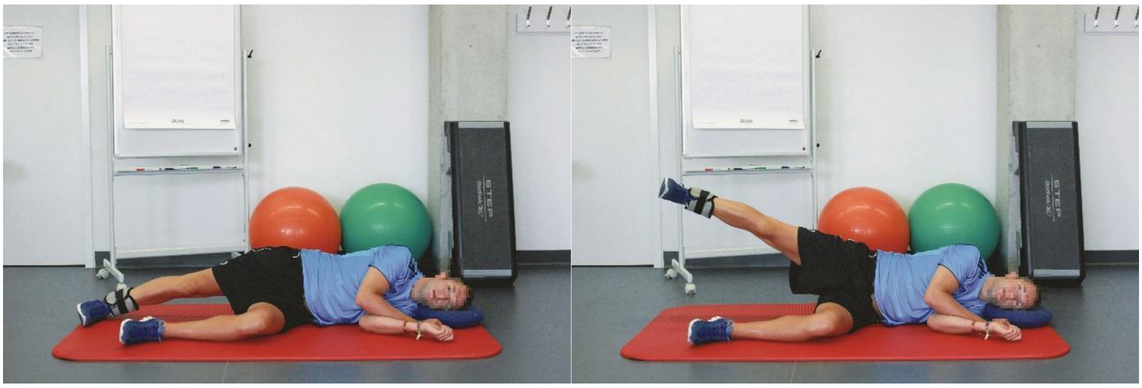

Hip 1: Abduction in sidelying position with the use of an ankle cuff weights

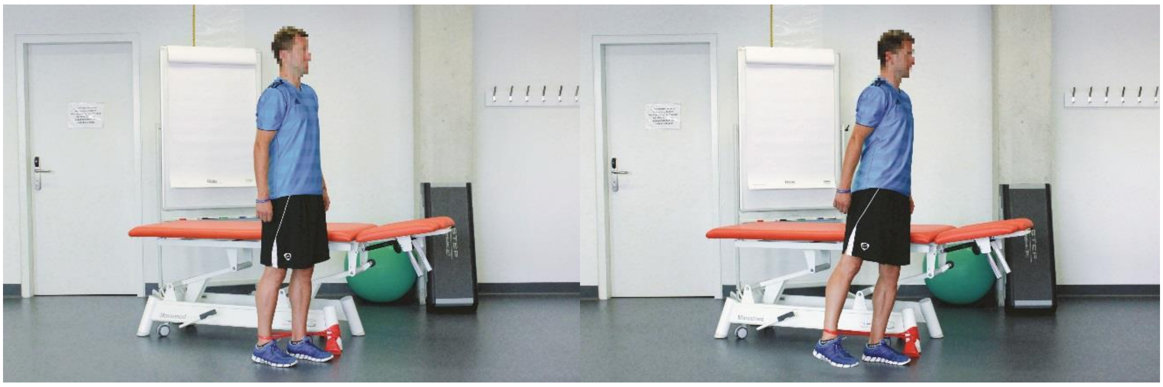

Hip 2: Abduction in standing position with the resistance of an elastic band

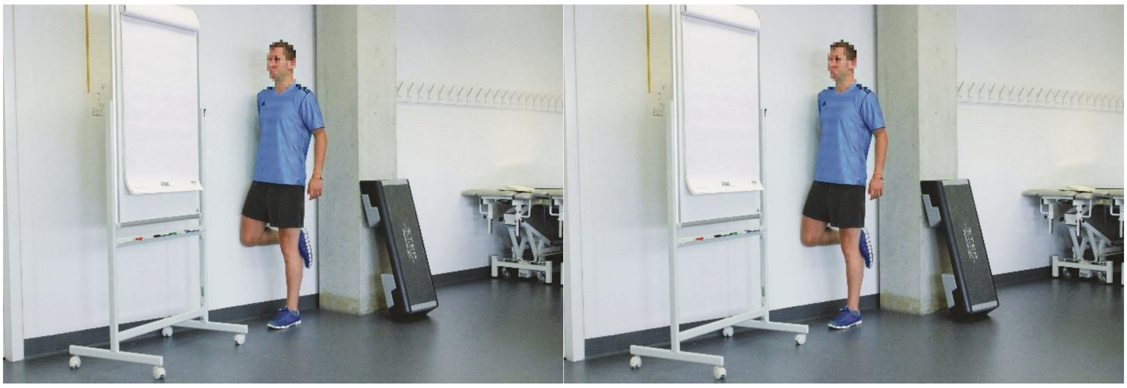

Hip 3: Standing wall isometric hip abduction

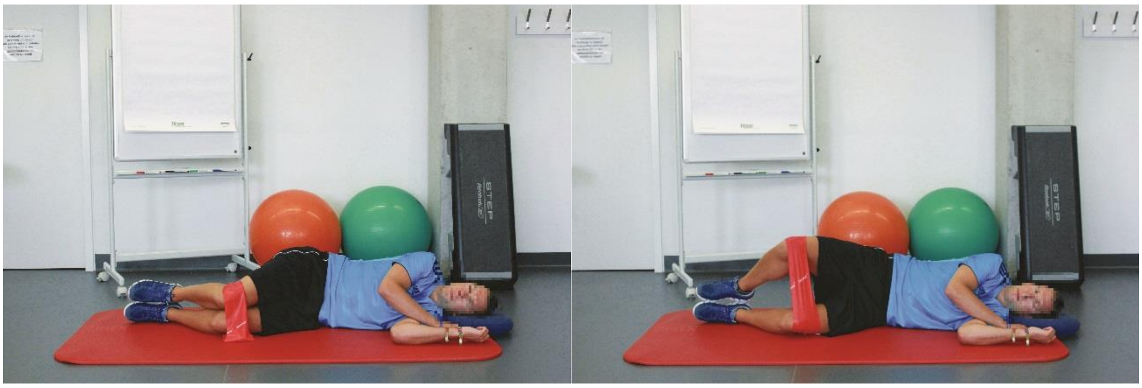

Hip 4: Clam in sidelying position with the resistance of an elastic band

Additional file 1

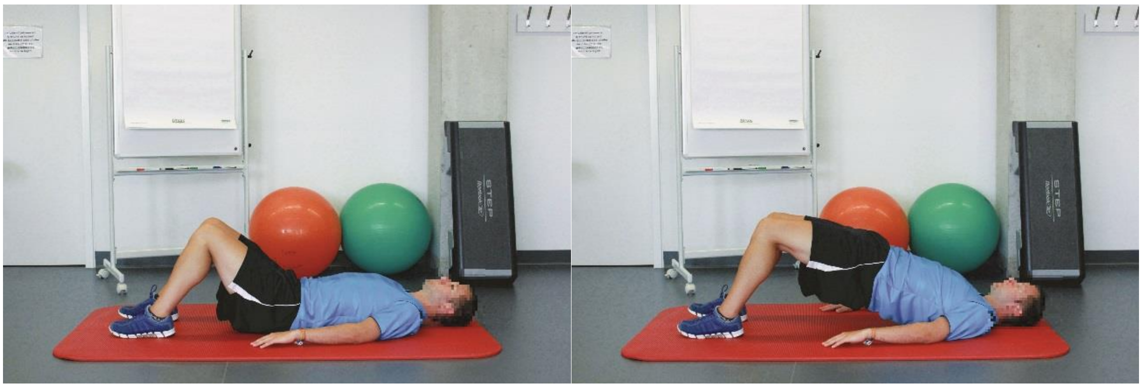

Hip 5: Bridging bilateral

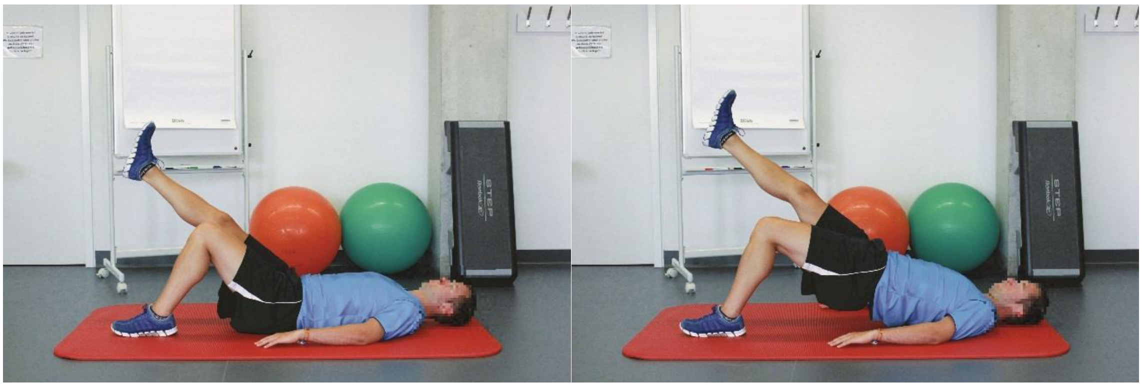

Hip 6: Bridging unilateral

Additional file 1

Exercise programme Part - Neuromuscular exercises

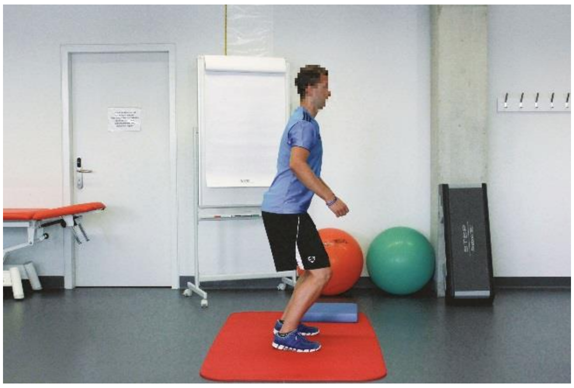

NM 1: Bilateral stance on a soft surface

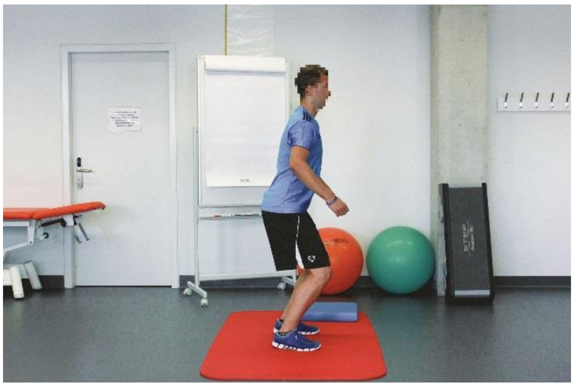

NM 2: Bilateral stance on a soft surface with eyes closed

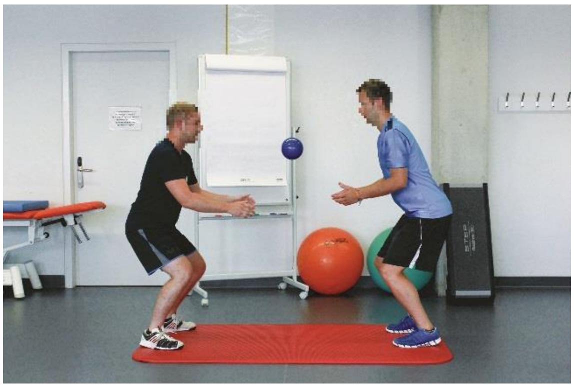

NM 3: Bilateral stance on a soft surface: two participants passing a ball back and forward

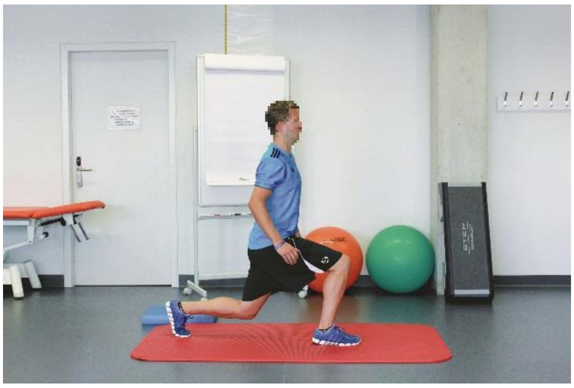

NM 4: Squat Lunge static on a soft surface

Additional file 1

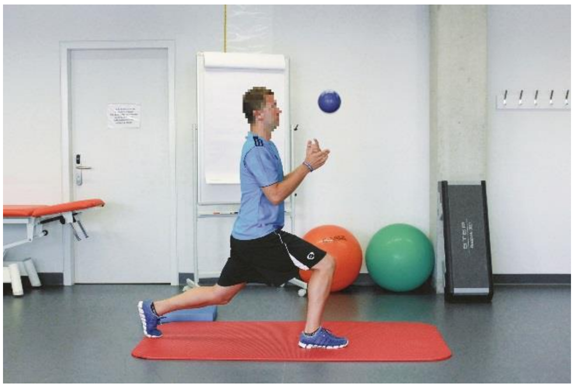

NM 5: Squat Lunge static on a soft surface and throwing and catching a ball

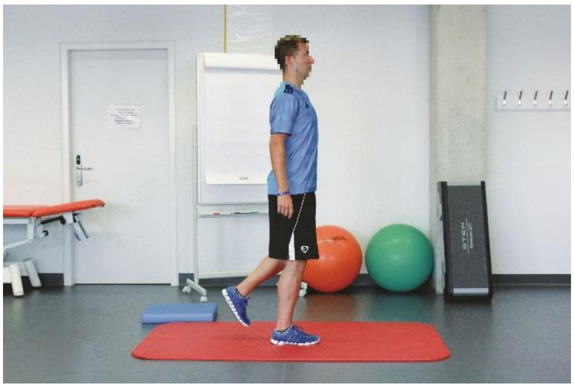

NM 6: Unilateral stance on a soft surface

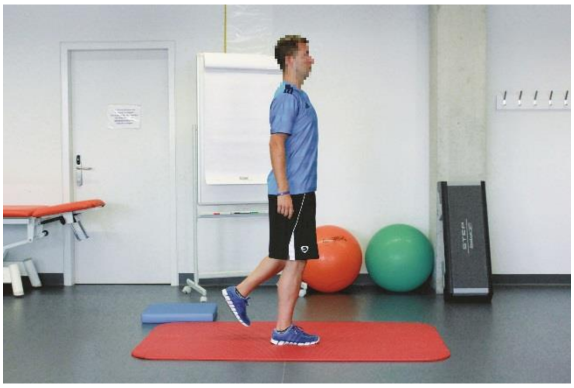

NM 7: Unilateral stance on a soft surface with eyes closed

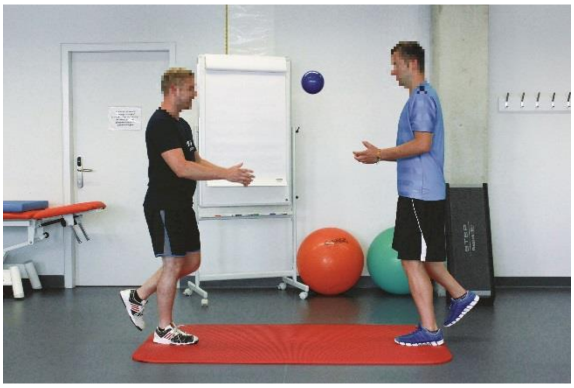

NM 8: Unilateral stance of a soft surface: two participants passing a ball back and forward
